# Supplementary material for: CLCA2: A Potential Guardian against Premature Senescence and Skin Aging
Source: Biomedicines. 2024 Mar 6;12(3):592. doi: 10.3390/biomedicines12030592 (PMC10968159; doi:10.3390/biomedicines12030592)
Supplement: Supplementary file 1 [file biomedicines-12-00592-s001.zip › biomedicines-2898248-supplementary.pdf]

**Supplementary Materials**

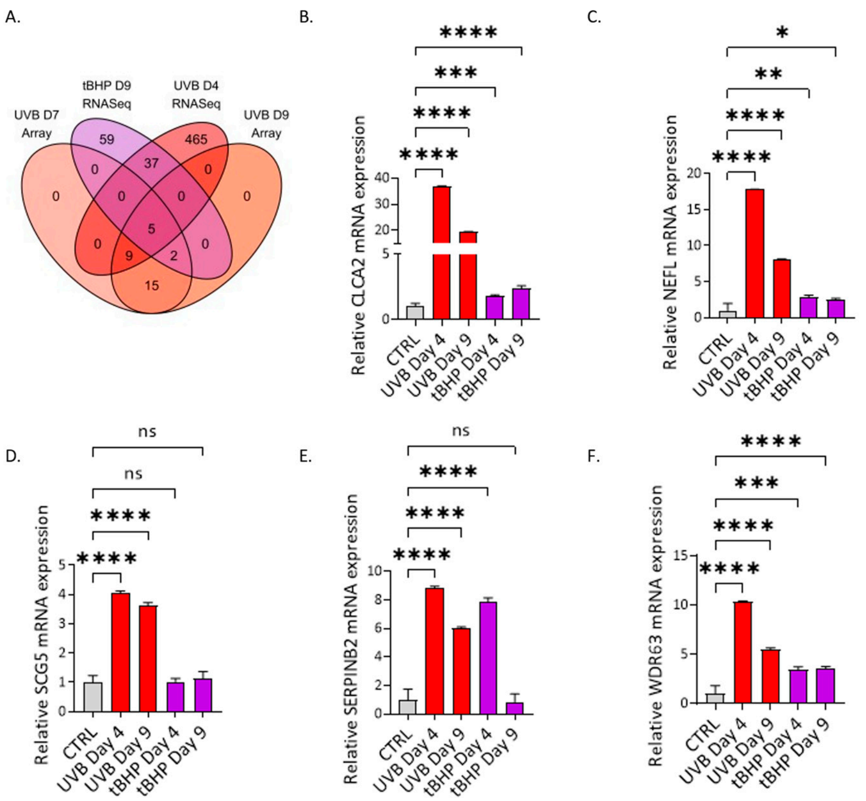


**Supplementary Figure S1.** *Candidate genes validation* A. Common genes from 4 data sets (tBHP Day 9 RNAseq, UVB Day 4 RNAseq, UVB Day 7 Array, UVB Day 9 Array) are shown as a Venn Diagram. mRNA expression from RT-qPCR in the candidates B. CLCA2, C. NEFL, D. SCG5, E.

SERPINB2, F. WDR63. Data represents mean values ± SD, N=3. In all graphics ns: non-significant, *p < 0.05, **p < 0.01, ***p < 0.001, ****p < 0.0001.

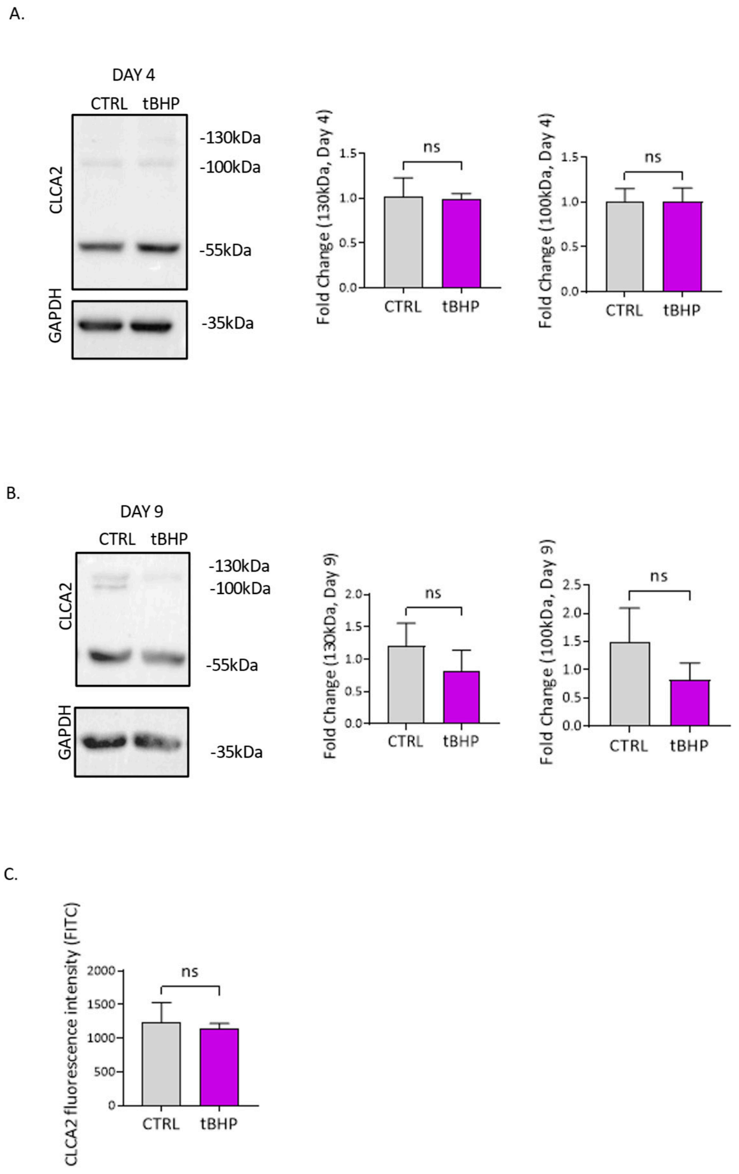


**Supplementary Figure S2.** *CLCA2 protein expression is not regulated by tBHP.* A. CLCA2 western

blot protein expression in tBHP model Day 4. B. CLCA2 western blot protein expression in tBHP model Day 9. C CLCA2 fluorescence intensity from FACS tBHP-induced senescent HDF (Day 9). Data represents mean values ± SD, N=3. In all graphics ns: non-significant, *p < 0.05, **p < 0.01, ***p < 0.001, ****p < 0.0001.

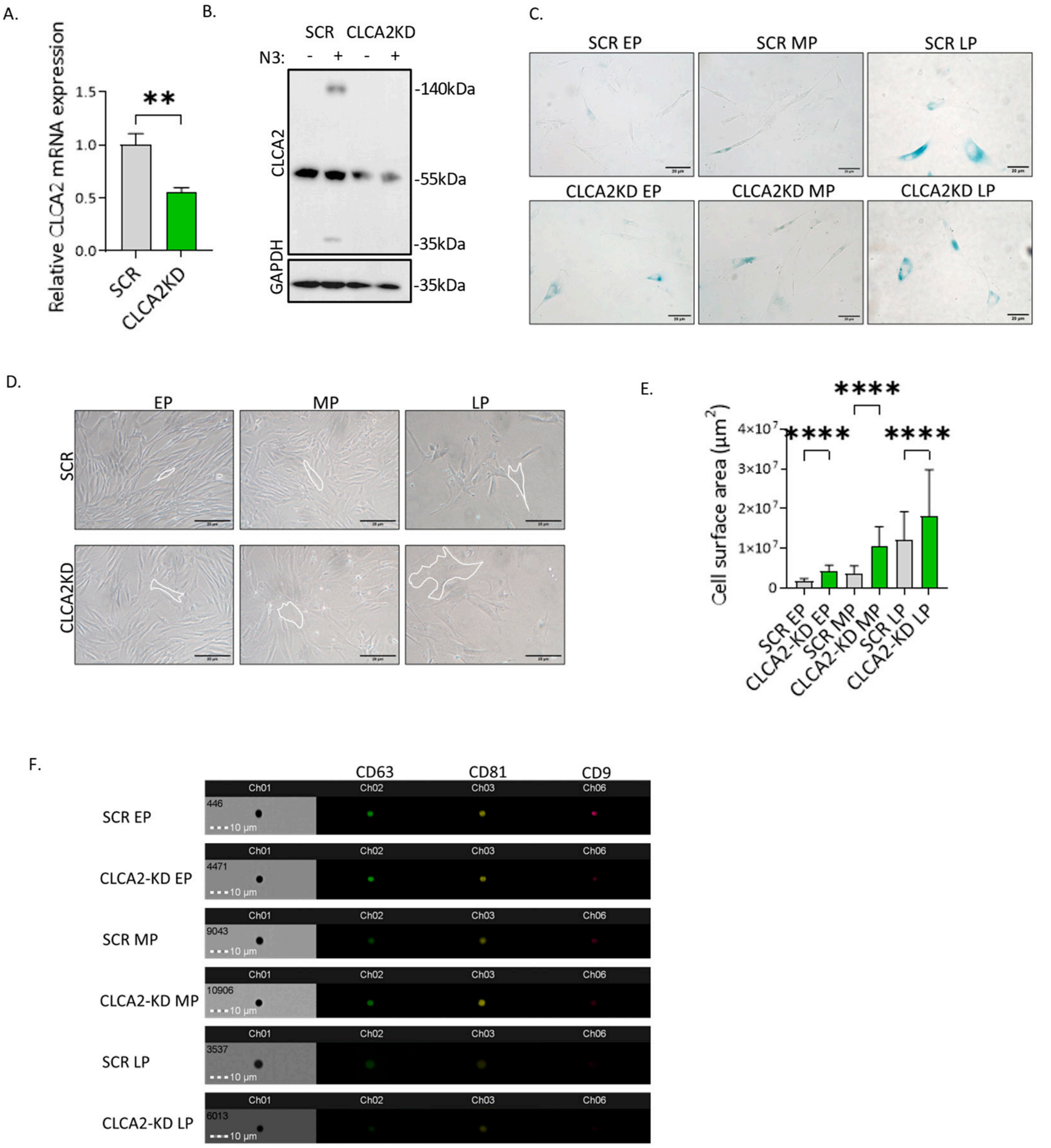


**Supplementary Figure S3.** *CLCA2KD HSDF show signs of premature senescence.* A. CLCA2 mRNA expression by RT-qPCR in SCR and CLCA2KD HDF. B. CLCA2 western blot protein expression in SCR and CLCA2KD untreated and N3 treated HDF (N=1). C. SA-β-gal staining pictures from SCR and CLCA2KD HDF. D. Representative pictures from SCR and CLCA2KD HDF at different passages. E. Measurement of cell surface area. F. CD81, CD63 and CD9 expression in EVs assessed by

Imagestream. Data represents mean values ± SD, N=3. In all graphics ns: non-significant, *$p < 0.05$, **$p < 0.01$, ***$p < 0.001$, ****$p < 0.0001$.

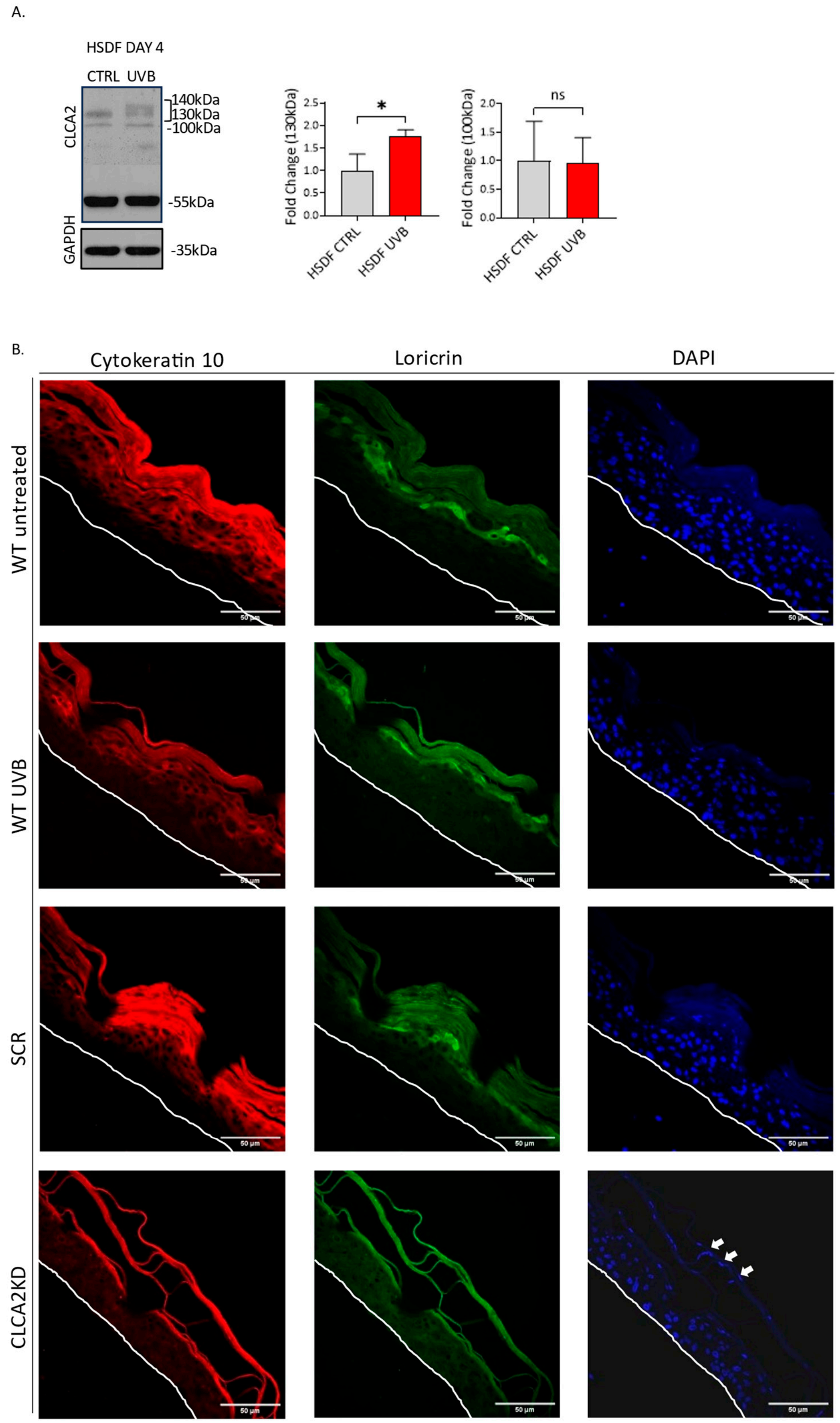


**Supplementary Figure S4.** *Epidermal differentiation markers in UVB and CLCA2KD SE.* A. CLCA2 protein expression by western blot in CTRL and UVB irradiated HSDF. B. Cytokeratin 10 and Loricrin immunofluorescence images from WT untreated, WT UVB, SCR and CLCA2KD SE. White arrows show nucleated cells. Data represents mean values ± SD, N=3. In all graphics ns: non-significant, *p < 0.05, **p < 0.01, ***p < 0.001, ****p < 0.0001.
